# Both diet and gene mutation induced obesity affect oocyte quality in mice

**DOI:** 10.1038/srep18858

**Published:** 2016-01-06

**Authors:** Yan-Jun Hou, Cheng-Cheng Zhu, Xing Duan, Hong-Lin Liu, Qiang Wang, Shao-Chen Sun

**Affiliations:** 1 College of Animal Science and Technology, Nanjing Agriculture University, Nanjing, 210095, China; 2 State Key Laboratory of Reproductive Medicine, Nanjing Medical University, Nanjing 210029, China

## Abstract

Obesity was shown to cause reproductive dysfunctions such as reduced conception, infertility and early pregnancy loss. However, the possible effects of obesity on oocyte quality are still not fully understood. In this study we investigated the effects of both diet and gene mutation induced obesity on impairments in mouse oocyte polarization, oxidative stress, and epigenetic modifications. Our results showed that high-fat diet induced obesity (HFD) and gene mutation induced obesity (ob/ob) could both impair oocyte meiotic maturation, disrupt spindle morphology, and reduce oocyte polarity. Oocytes from obese mice underwent oxidative stress, as shown by high DHE and ROS levels. Abnormal mitochondrial distributions and structures were observed in oocytes from obese groups of mice and early apoptosis signals were detected, which suggesting that oxidative stress had impaired mitochondrial function and resulted in oocyte apoptosis. Our results also showed that 5 mC levels and H3K9 and H3K27 methylation levels were altered in oocytes from obese mice, which indicated that DNA methylation and histone methylation had been affected. Our results showed that both HFD and ob/ob induced obesity affected oocyte maturation and that oxidative stress-induced early apoptosis and altered epigenetic modifications may be the reasons for reduced oocyte quality in obese mice.

The prevalence of obesity among women of reproductive age is increasing. Based on its pathogenesis, obesity is categorized as either diet-induced, gene mutation induced, or other disease-induced obesity. Gene mutation obesity (ob/ob) and high-fat diet induced obesity (HFD) have been shown to be associated with reproductive problems, such as infertility, embryo implantation failure, abortion, fetal congenital abnormalities, adult offspring adiposity, and metabolic dysfunction^1^,^2^,^3^,^4^. A previous report emphasized that optimal maternal weight and nutrition prior to conception were important for the offspring, as maternal obesity had adverse effects as early as the oocyte and preimplantation embryo stages^5^. This proposal was confirmed in that the increased pregnancy failure rate in women with obesity returned to a normal rate if donor oocytes were used rather than autologous oocytes^6^.

Obesity impairs ovary function as shown by reduced numbers of corpora lutea and expression of steroidogenic acute regulatory enzymes and enhanced follicular atresia along with apoptosis^7^. Additionally, small oocyte^8^ and reduced numbers of oocytes^9^ have been found in ob/ob mice. Previous studies of HFD induced obesity showed that there were more apoptotic ovarian follicles, fewer mature oocytes, and smaller fetuses for mice with obesity^10^ and oocytes from HFD mice had abnormal fragmented spindles and clustered chromosomes^11^. Additionally, lipid accumulation, ER stress, mitochondrial dysfunction, and apoptosis were markedly increased in the ovarian cells of mice that were fed a high-fat diet and these mice had increased anovulation and reduced *in vivo* fertilization rates^12^. Reduced blastocyst survival rates and abnormal embryonic cellular differentiation were also found in HFD induced obesity mice^13^. However, there has been little work on the effects of obesity, particularly gene mutation induced obesity, on oocyte quality and its possible associated mechanisms.

Oxidative stress, which is defined as an imbalance between pro-oxidant and antioxidant capacity, has been implicated in suboptimal reproductive performance^14^. Women with obesity have high circulating levels of catalase and glutathione peroxidase enzyme activities, which indicates that their bodies are suffered from oxidative stress^15^. Oocyte quality is also affected by oxidative stress, and lower fertility rates among cigarette smokers or in association with high levels of alcohol consumption have been linked to increased ROS synthesis^16^,^17^. Moreover, oxidative stress has been shown to be associated with changes in mitochondrial activity. Research on type 1 diabetics showed that abnormal mitochondrial structures and functions in oocytes were associated with poor fertilization rates and abnormal embryo development in mice^18^. Furthermore, it was shown that cells that were suffering from oxidative stress resulted in their apoptosis, which may have resulted from impaired mitochondrial function and the activation of apoptotic factors in mouse kidneys^19^.

Epigenetic phenomena are heritable changes in gene expression that do not involve alterations in nucleotide sequences. These modifications include chromatin methylation and post-translational modifications of histones or other chromatin-associated proteins. DNA methylation is a key epigenetic modification that is essential for normal embryonic development^20^, and epigenetic information linked to environmental factors participates in the early stages of development^21^. A previous study showed that DNA methylation was altered in the oocytes of HFD induced obesity mice and in the oocytes and livers of their offspring^22^. Moreover, changes in histone modifications are key components of an epigenetic network that controls energy homeostasis^23^. For example, H3K9 and H3K27 play important roles in regulating gene expression in mitotic cells^24^. H3K9 methylation is also an epigenetic marker of parental genome origin during early preimplantation embryo development^25^.

In this study, we investigated maturation capability, spindle morphology, changes in oocyte polarization, oxidative stress levels, mitochondrial distributions and morphologies, and any epigenetic modifications in oocytes from both HFD and ob/ob mice. We compared the effects of both diet-induced and gene mutation-induced obesity on oocyte quality, and investigated the mechanisms possibly involved in these oocytes.

## Results

### Both high fat diet and gene mutation induced obesity affect mouse oocyte maturation

**Figure 1:**
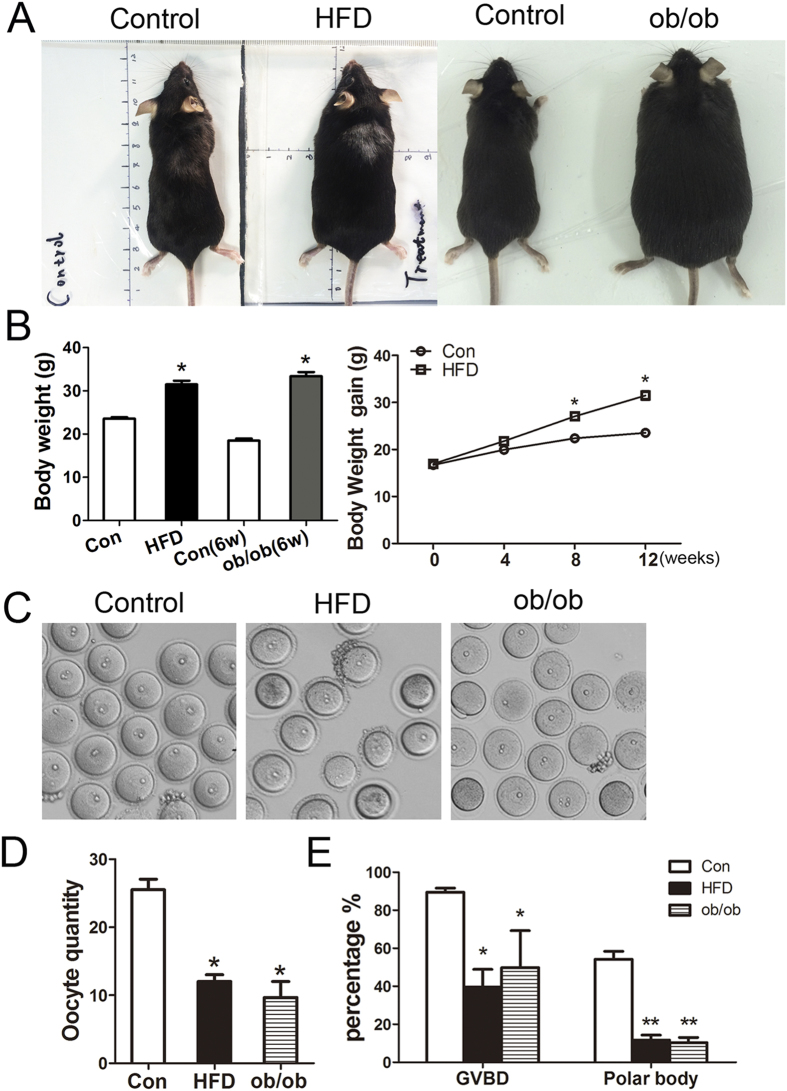
Obesity effects on mouse body weight and oocyte maturation. (**A**,**B**) The body weights of HFD and ob/ob mice were significantly greater than those of control mice. (**C**) HFD and ob/ob mice had dark, smaller oocytes. (**D**) The numbers of oocytes from both obesity groups were reduced. (**E**) The proportions of GVBD in oocytes and polar body extrusion rates were reduced for oocytes from HFD and ob/ob induced mice with obesity.

The body weights of HFD mice (31.48 ± 0.87 g, n = 60) were significantly greater than those of control mice (23.54 ± 0.34 g, n = 60) and 6-week-old ob/ob mice were also significantly heavier (33.37 ± 0.98 g, n = 60) than control (6 w) mice (19.16 ± 0.49 g, n = 60) (Fig. 1A,B). We then obtained GV oocytes from these four groups to examine oocyte maturation.

Our results showed that the GV oocyte number we collected from the ovaries of HFD mice (12.00 ± 1.00, n = 12) and from ob/ob mice (9.67 ± 2.33, n = 12) were significantly reduced as compared to those from the ovaries of control mice (25.5 ± 1.57, n = 12) (Fig. 1C,D). The proportions of oocytes with germinal vesicle breakdown (GVBD) and polar body extrusion are shown in Fig. 1E. Most oocytes underwent GVBD in the control group after culture for 3 h. However, a significantly reduced GVBD rate was found for oocytes in the obesity groups: 89.46 ± 3.26% (n = 124) for controls *vs.* 39.45 ± 9.41% (n = 82) for HFD and 49.83 ± 14.78% (n = 37) for ob/ob (*P* < 0.05). Oocyte maturation was also significantly affected by obesity: 54.17 ± 4.31% (n = 128) of control oocytes matured to metaphase II (MII) after culture for 12 h *in vitro* compared with 11.26 ± 2.97% (n = 51) of HFD oocytes and 10.37 ± 2.74% (n = 44) of ob/ob oocytes (*P* < 0.05).

### Obesity disrupts meiotic spindle morphology and reduces oocyte polarity

**Figure 2:**
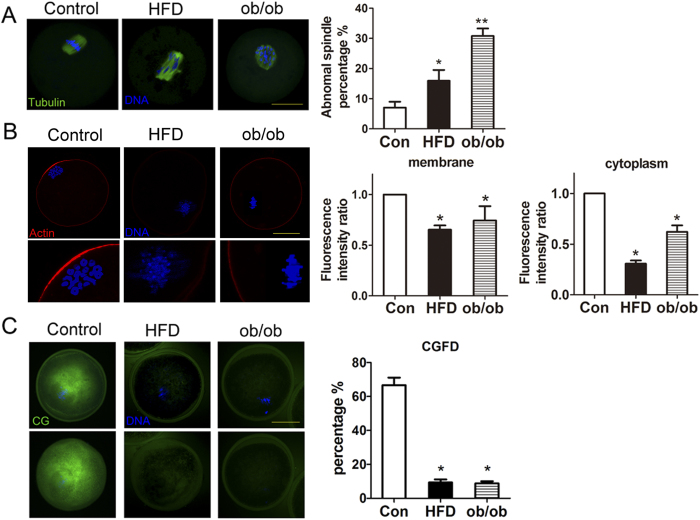
Obesity disrupts meiotic spindle morphology and alters oocyte polarization. (**A**) Oocytes from both groups of mice with obesity had misaligned chromosomes and disrupted spindle morphologies and the rates of abnormal spindle morphology were higher than that of control oocytes. (**B**) Actin fluorescence intensity at the plasma membrane and in the cytoplasm was lower than that in control MI oocytes. (**C**) CGs were uniformly localized at the plasma membrane and CG signals were weaker in oocytes from mice with obesity.

We next examined spindle morphology and the localization of actin filaments to assess any oocyte quality changes. The majority of MI oocytes in the control group had well-aligned chromosomes and normal spindle morphologies. However, a higher proportion of oocytes had misaligned chromosomes and disrupted spindle morphologies in the obesity groups. The abnormal rates of spindle morphology were: 7.07 ± 1.91% (n = 115) for controls vs. 15.91 ± 3.62% (n = 155) for HFD oocytes and 30.77 ± 2.51% (n = 127) for ob/ob oocytes (*P* < 0.05; Fig. 2A).

We next examined actin cap formation and the distributions of cortical granules (CGs) at MІ stage. For metaphase I (MI) oocytes from HFD and ob/ob mice, actin fluorescence intensity at the plasma membrane and in the cytoplasm was significantly lower (0.65 ± 0.04 for membrane actin and 0.31 ± 0.03 for cytoplasmic actin in HFD oocytes vs. 0.74 ± 0.14 for membrane actin and 0.62 ± 0.06 for cytoplasmic actin in ob/ob oocytes) than that in control MI oocytes (1.00 ± 0.00 for membrane actin and 1.00 ± 0.00 for cytoplasmic actin) (*P* < 0.05; Fig. 2B). We also examined CGs, which is an indicator of oocyte polarity. In control oocytes, CGs were clearly located under the plasma membrane and absent in areas close to chromosomes, whereas CGs were uniformly localized at the plasma membrane and CG signals were weaker in oocytes from mice with obesity. The formation of cortical granules-free domain (CGFD) was analyzed as a further feature of oocyte polarity. The proportions of CGFD were decreased in HFD oocyte (9.33 ± 1.76%, n = 65) and in ob/ob oocyte (8.75 ± 1.25%, n = 49), while it was 66.67 ± 4.41% (n = 76) in control oocyte (*P* < 0.05; Fig. 2C).

### Obesity induces oxidative stress in mouse oocytes

**Figure 3:**
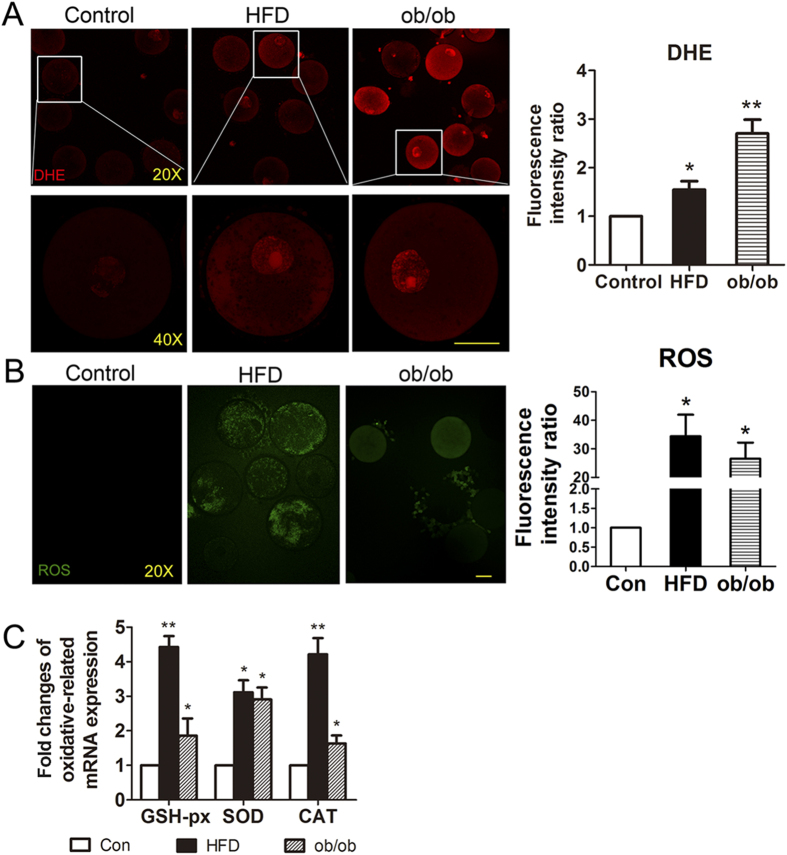
Obesity results in oocyte oxidative stress. (**A**) DHE expression was increased in oocytes from both obesity groups as were the fluorescence intensity ratios for oxidative stress in these oocytes. (**B**) ROS levels determined by fluorescent staining and the fluorescence intensity ratios of ROS. (**C**) Fold-changes in oxidative stress related genes’ mRNA expression were altered in oocytes from both HFD and ob/ob induced mice with obesity.

Oxidative stress is a cellular response to noxious external stimuli. To determine whether obesity induced oxidative stress in diet and gene mutation induced mice with obesity, we first investigated dihydroethidium (DHE) and reactive oxygen species (ROS) levels in GV oocytes. This showed that DHE and ROS levels were both increased in GV oocytes from the two obesity groups. We then assessed the fluorescence intensity ratios of oxidative stress signals in oocytes from the HFD and ob/ob groups. The fluorescence intensity ratios of DHE in GV oocytes from obesity groups (1.55 ± 0.17 for HFD mice and 2.70 ± 0.28 for ob/ob mice) were significantly higher than those of controls (*P* < 0.05; Fig. 3A). And the fluorescence intensity ratios of ROS in GV oocytes from both obesity mice (34.38 ± 7.56 for HFD mice and 26.53 ± 5.66 for ob/ob mice) were significantly increased compared to controls (*P* < 0.05; Fig. 3B).

Next, we examined mRNA expression levels of oxidative stress related genes. This showed that the mRNA levels of the genes for anti-oxidative enzymes, including GSH-Px, SOD, and CAT, were significantly increased in HFD and ob/ob oocytes as compared to those in controls (Fig. 3C). The relative mRNA levels as compared to those in controls were: 4.42 ± 0.32 for GSH-Px, 3.11 ± 0.35 for SOD, and 4.21 ± 0.47 for CAT in HFD oocytes; and 1.86 ± 0.50 for GSH-Px, 2.91 ± 0.35 for SOD, and 1.63 ± 0.23 for CAT in ob/ob oocytes (Fig. 3C).

### Obesity disrupts oocyte mitochondrial distributions and induces early apoptosis

**Figure 4:**
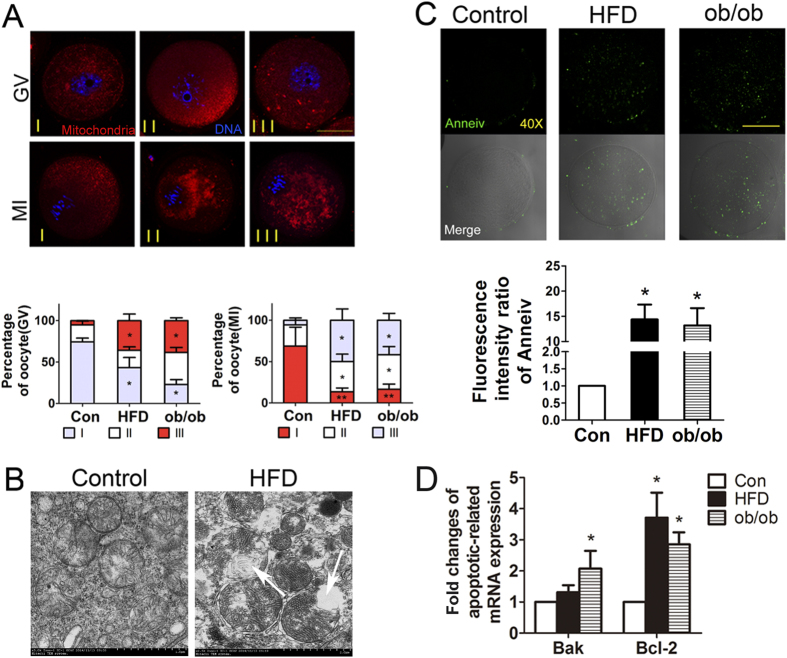
Obesity disrupts distributions and morphology of mitochondria and induces early oocyte apoptosis. (**A**) Mitochondria distribution patterns in oocytes at the GV and MI stages. The distribution patterns of mitochondria in oocytes from HFD and ob/ob mice were primarily clustered. (**B**) Transmission electron microscopy images showing mitochondrial morphologies in oocytes from HFD and control mice. Arrows indicate abnormal mitochondria that lack cristae and have broken membranes. (**C**) Early apoptosis signals in oocytes were assessed using Annexin V/PI kits. Oocytes from HFD and ob/ob mice had bright fluorescent signals. (**D**) Bak and Bcl-2 mRNA expressions in oocytes. Both obesity groups had higher fold-changes in mRNA expressions.

To confirm that there were impairments in mitochondria in the oocytes from mice with obesity, we examined the distributions and morphologies of mitochondria in mouse oocytes. Based on the results of a previous report^26^, GV oocytes were categorized into three groups based on their mitochondria distributions: І, perinuclear and surrounding the GV (normal); II, homogenous and throughout the entire ooplasm; and III, clustered in the cytoplasm (abnormal) (Fig. 4A). For controls (n = 100), the mitochondria distributions in GV oocytes were perinuclear (74.47 ± 4.47%), homogenous (20.39 ± 4.61%), and clustered (5.13 ± 0.13%). In contrast, the distribution patterns of mitochondria in oocytes from HFD (n = 62) and ob/ob mice (n = 62) were clustered (35.76 ± 7.99%), with perinuclear (43.40 ± 12.15%), and homogenous (20.83 ± 4.17%) for HFD mice, and clustered (38.46 ± 3.44%), with perinuclear (23.08 ± 5.75%), and homogenous (38.46 ± 6.12%) for ob/ob mice. Compared with oocytes from control mice, the clustered patterns of mitochondrial localization in the obesity groups were significantly more frequent (*P* < 0.05; Fig. 4A).

After maturation for 9 h, the distributions of mitochondria in MI stage oocytes were categorized as previously described (17): polarity (I), surrounding a chromosome (II), and cluster like little clumps (III) (Fig. 4A). The oocytes from HFD (50.00 ± 13.64%, n = 86) and ob/ob (41.66 ± 8.26%, n = 30) mice had significantly higher proportions of clustered mitochondria as compared to control oocytes (5.59 ± 2.74%, n = 92; *P* < 0.05), while polarity morphology percentages were significantly reduced in the two obesity groups (68.69 ± 22.98% for controls *vs.* 13.64 ± 4.54% for HFD and 16.67 ± 6.21% for ob/ob; *P* < 0.05; Fig. 4A).

Next, we used transmission electron microscopy to examine for any changes in mitochondrial morphology. This showed that the oocyte mitochondria from mice on the high fat diet had broken membranes, fewer cristae, increased swelling, and more vacuoles (Fig. 4B), which was consistent with previous results^11^.

These results indicated that HFD and ob/ob obesity had profound effects as early as the oocyte meiosis stage and might interfere with oocyte metabolism due to induced oxidative stress and impaired mitochondria.

We also examined for early apoptosis signals in oocytes. This showed that Annexin V/PI fluorescence signals were increased in oocytes from HFD (14.38 ± 2.94) and ob/ob (13.19 ± 3.43) mice with obesity as compared to those in controls (1.00 ± 0.00) (*P* < 0.05; Fig. 4C). Furthermore, we examined Bak and Bcl-2 mRNA expressions in oocytes, which results confirmed the results of Annexin V/PI staining. The fold-changes in Bak and Bcl-2 mRNA expression of ob/ob group and the Bcl-2 mRNA expression of HFD group were increased (1.31 ± 0.23, p > 0.1 for Bak and 3.71 ± 0.80, p < 0.05 for Bcl-2 of HFD group; 2.07 ± 0.57, p < 0.05 for Bak and 2.85 ± 0.39, p < 0.05 for Bcl-2 of ob/ob group) compared to the controls (Fig. 4D).

### Obesity affects epigenetic modification levels in mouse oocytes

**Figure 5:**
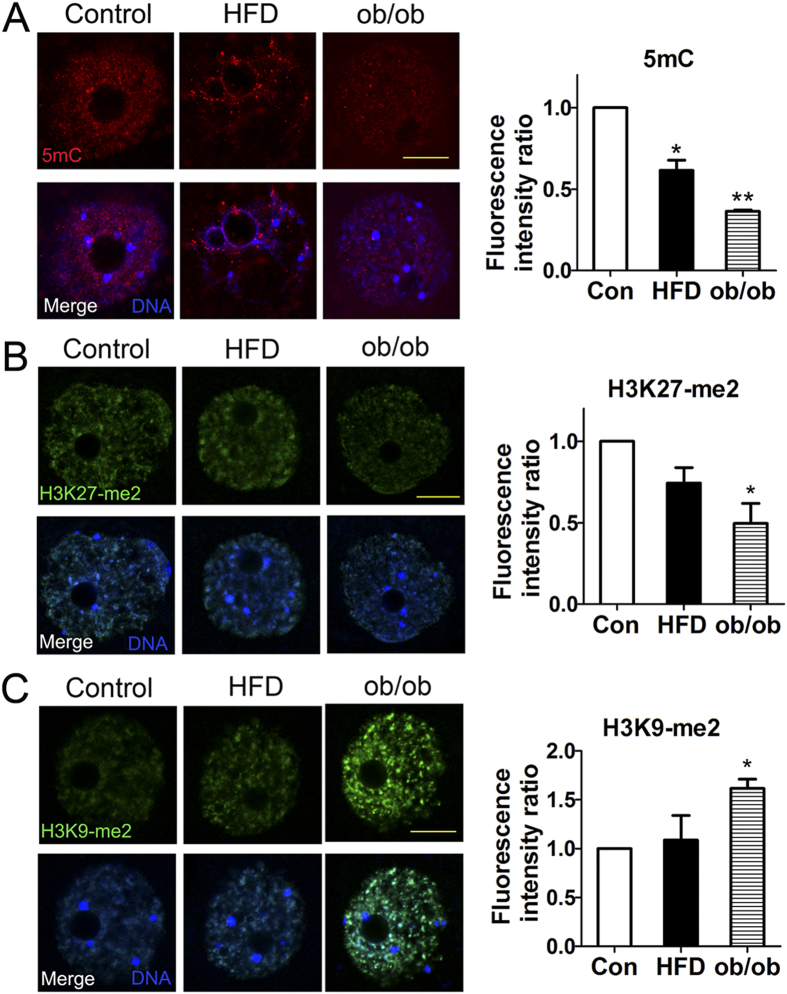
Obesity affects epigenetic modifications in oocytes. (**A**) 5 mC genomic contents. 5 mC expression levels in oocytes from HFD and ob/ob mice with obesity were reduced compared to that in controls. (**B**) H3K27-me2 genomic contents. The H3K27-me2 fluorescence intensity ratio was reduced in oocytes from both HFD and ob/ob induced mice with obesity as compared to that in controls. (**C**) H3K9-me2 genomic contents. The H3K9-me2 fluorescence intensity ratio was increased in oocytes from both HFD and ob/ob induced mice with obesity as compared to that in controls.

Two typical epigenetic modifications are well known to significantly influence DNA function: DNA methylation and histone modifications. Then we attempted to examine the epigenetic modifications in the NSN (no Hoechst-positive rim surrounding the nucleolus) GV oocytes, since NSN stage oocytes had a higher gene transcription activity which could reflect the early methylation levels^27^. We assessed the levels of 5 mC in GV oocytes, which showed that 5 mC expression levels both in HFD (0.61 ± 0.06) and ob/ob (0.36 ± 0.01) oocytes were reduced as compared to controls (1.00 ± 0.00) (*P* < 0.05; Fig. 5A). We also found that the H3K27-me2 fluorescence intensity ratios were reduced in ob/ob (0.49 ± 0.12, p < 0.05) oocytes as compared to controls (1.00 ± 0.00; Fig. 5B) but not in HFD oocytes (0.74 ± 0.09, p > 0.1); whereas the H3K9-me2 fluorescence intensity ratios were increased in ob/ob (1.62 ± 0.10, p < 0.05) oocytes as compared to controls (1.00 ± 0.00; Fig. 5C) but not in HFD oocytes (1.09 ± 0.17, p > 0.1).

## Discussion

The burdens of obesity and the associated health problems have become global issues. Our results suggest that oocytes from both HFD and ob/ob mice with obesity had low rates of maturation, disrupted spindle morphologies, and reduced oocyte polarization. We also found that oocytes from HFD and ob/ob mice with obesity were suffering from oxidative stress, had alterations in mitochondria distributions, and experienced early apoptosis. Additionally, these oocytes had changes in DNA cytosine methylation and the methylation patterns of H3K9-me2 and H3K27-me2.

A previous study demonstrated that oocytes from HFD induced mice with obesity were smaller and had delayed maturation as compared to those from control mice^10^. In our study, we found that the numbers of oocytes from both HFD and ob/ob induced mice with obesity were reduced as compared to those from normal mice, and the maturated proportions and polar body extrusion percentages were reduced as compared to those oocytes from control mice. Abnormal spindles can impair oocyte meiosis and suppress polar body extrusion^28^. Other studies showed that the abnormal spindle morphologies of oocytes from HFD induced mice with obesity and diabetic model mice were increased^11^,^18^. We also found high percentages of abnormal spindle morphologies in the oocytes from both HFD and ob/ob induced mice with obesity. Moreover, during the late metaphase I stage, cortical granules (CGs) were lost in the region overlying the chromosomes and microfilaments were enriched under the membrane near the spindle to form an actin cap, which are features of oocyte polarity formation^29^. Our results showed that these two features were lost in most oocytes from mice with obesity, which further confirmed our hypothesis that HFD and ob/ob induced obesity both affected oocyte quality. Previous work showed that the fertility of mice with obesity was reduced which was due to the early embryonic loss in high fat diet induced obesity mice^11^. Our results, together with previous reports indicated that the infertility in mice with obesity might be due to the low quality of oocytes, which was reflected by the disrupted subcellular structures like cytoskeleton, mitochondria or chromosomes.

To determine a possible mechanism for how obesity affected oocyte quality, we investigated oxidative stress, apoptosis, and epigenetic modifications. HFD induced maternal obesity has been shown to be associated with increased ROS generation in different tissues^30^,^31^. Obesity also resulted in oxidative DNA damage and related genes’ expression changes in breast tissue^32^. In this study, we showed that oocytes were suffering from oxidative stress and that anti-oxidative gene mRNA expression levels were increased in the oocytes from both HFD and ob/ob induced mice with obesity. Impairments in mitochondria were shown to be related to oxidative stress and apoptosis^5^, and excessive nutrient intake was associated with poor reproductive outcomes in women with obesity and mice and their early embryo mitochondrial metabolism exhibited alterations^33^,^34^.

Other previous work indicated that this abnormal distribution pattern of mitochondria was related to apoptosis^7^, and HFD induced mice with obesity had fewer follicles and more apoptotic granulosa cells in their follicles as compared to normal mice^8^. In this study, we found that the oocytes from both HFD mice with obesity and ob/ob mice with obesity had higher proportions of the clustered distribution of mitochondria, early apoptosis signals, and higher levels of apoptosis related genes’ mRNA expression as compared to the oocytes from control mice. This indicated that similar to other cell types, oocytes from mice with obesity were also suffering from oxidative stress and early apoptosis. This may be one reason for the low quality of the oocytes from mice with obesity.

5 mC genomic levels are related to the metabolism and proliferation of cells^35^,^36^. In a study of oocytes and embryos, it was confirmed that the maternal DNA methylation pattern was maintained until the 16-cell stage^37^. Other reports showed that H3K27 was related to lipid metabolism^38^, and that H3K9 modulation in chromatin might be a new target for treating obesity and metabolic syndrome^39^. Studies of type 2 diabetes mellitus (T2DM) and HFD induced obesity demonstrated that DNA methylation was changed to promote genes’ expression related to mitochondrial biogenesis and function^4^. In our study, we found that the histone modification H3K9-me2 was enhanced, while the DNA methylation of 5 mC and histone modification H3K27-me2 were reduced in oocytes from both HFD and ob/ob mice with obesity. These results indicated that epigenetic modifications were altered in both HFD and ob/ob oocytes, which may have been another reason for the low quality of oocytes from mice with obesity.

In conclusion, we found that both HFD and ob/ob induced obesity in mice resulted in low oocyte quality, and that oxidative stress, early apoptosis, and epigenetic modification alterations might have been reasons for this phenomenon.

## Material and methods

### Ethic statement

Animal care and use were conducted in accordance with the guidelines of Nanjing Agricultural University, China. Mice were housed in a temperature-controlled room with proper darkness-light cycles, fed with a regular diet, and maintained under the care of the Laboratory Animal Unit, Nanjing Agricultural University, China. The mice were sacrificed by cervical dislocation. This study was specifically approved by the Institutional Animal Care and Use Committee, Nanjing Agricultural University, China.

### Animals and oocyte harvesting

Female C57BL-6 mice (4-week-old) were purchased from Nanjing Medical University (Jiangsu, China) and housed under conditions of constant temperature (24 °C ± 2 °C) and light: dark (12 h: 12 h). They had unrestricted access to food and water and were maintained under the care of the Laboratory Animal Unit, Nanjing Agricultural University. Mice were fed either a control diet (MD12031, 10 g/100 g fat, Mediscience Ltd., China) or a high-fat diet (HFD, MD12032, 45 g/100 g fat, Mediscience Ltd., China) for 12 weeks. Six-week-old B6.Cg-Lep^ob^/JNju (*ob/ob*) mice (C57bl6 background) were purchased from Nanjing University (Jiangsu, China) and were fed the control diet and housed as described above. After the experimental period, 9 mice were weighed and sacrificed by cervical dislocation without gonadotropins, their ovaries were removed and weighed, and germinal vesicle (GV) oocytes were harvested for the analysis of oocyte number and the proportions of oocyte meiotic maturation *in vitro*. The other mice were sacrificed with gonadotropins, and GV oocytes harvested for the other examinations.

GV oocytes were harvested from the ovaries of control mice and obesity mice. And then the GV oocytes which were without cumulus cells were cultured in M16 medium (Sigma-Aldrich) under paraffin oil at 37 °C and 5% CO_2_ (v/v). After 4 hours culture, the proportions of germinal coticule breakdown (GVBD) were examined. The oocyte polarizations were analyzed at late MІ stage (9 h), and the proportion of oocyte maturation were examined at MII stage. These samples were used for the following experiments.

### Antibodies and chemicals

Mouse monoclonal anti-5 mC antibody was purchased from Abcam (Cambridge, UK). Rabbit polyclonal anti-H3K9-me2 antibody was from Upstate Chemicals (Billerica, MA). Rabbit monoclonal anti-H3K27-me2 antibody was from Cell Signaling Technology (Beverly, MA). Hoechst 33342, anti-α-tubulin-FITC, and phalloidin-TRITC (actin) were from Sigma-Aldrich (St. Louis, MO, USA). Mito Tracker Red used to stain mitochondria was from Invitrogen (Molecular Probes Eugene, OR, USA). Alexa Fluor 488 and 568 goat anti-rabbit and anti-mouse secondary antibodies were from Invitrogen (Carlsbad, CA, USA).

Reactive oxygen species (ROS) and dihydroethidium (DHE) were determined using kits(S0033 for ROS and S0063 for DHE)from Beyotime (Beyotime Lot. China), according to the manufacturer’s instructions.

### RNA isolation and real-time quantitative PCR analysis

Total oocyte RNA was isolated using a Dynabeads® mRNA DIRECT™ Purification Kit (Life Technologies Lot. USA), according to the manufacturer’s instruction. A first cDNA strand was synthesized using a Reverse Transcription System A3500 (Promega, USA). Real-Time quantitative PCR was done using SYBR Premix Ex Taq (Takara) in a reaction volume of 20 μL and conducted using a fast real-time PCR system (ABI Step One Plus, Life Technology Lot. USA). Primers were synthesized by Invitrogen Lot. Primer sequences are given in Supporting Information (Supporting Information Table S1). Data were analyzed by ΔΔCt method, and the GAPDH was used as the housekeeper gene.

### Confocal microscopy

For single staining of actin, CGs, or α-tubulin, oocytes were fixed in 4% paraformaldehyde in phosphate-buffered saline (PBS) for 30 min at room temperature. Then they were transferred to membrane permeabilization solution (0.5% Triton X-100) for 20 min. Oocytes were incubated in blocking buffer (1% BSA-supplemented PBS) for 1 h overnight at 4 °C or 4 h at room temperature with 10 μg/ml of Phalloidin-TRITC, 100 μg/ml of Lectin-FITC, or anti-α-tubulin-FITC antibody (1:200). After 3 washes, they were co-stained with Hoechst 33342 (10 μg/mL in PBS) for 10 min.

For staining of mitochondria, oocytes were cultured in M16 medium that contained 200 nM Mito Tracker Red (Molecular Probes Eugene, OR), at 37 °C for 30 min. After three washes in PBS containing 1% bovine serum albumin (BSA), oocytes were fixed with 4% paraformaldehyde in PBS for 30 min and then transferred to a membrane permeabilization solution (0.5% Triton X-100) for 20 min. After three washes, oocytes were co-stained with Hoechst 33342 for 10 min, then samples were analyzed by confocal microscopy. Reactive oxygen species (ROS) and Superoxide (DHE) production was measured by fluorescent microscopy and flow cytometry using the ROS and superoxide detection kit (Beyotime Biotechnology, China). Oocytes were stained for 30 min at 37 °C in the dark with 1 μM of ROS and superoxide-sensitive fluorescent dyes and then washed once with 1× wash buffer. The oocytes were treated with 5 mM ATP for 30 min. Fluorescence signals were subsequently detected with excitation at 488 nm and emission at 525 nm for the detection of ROS and with excitation at 550 nm and emission at 620 nm for the detection of superoxide. Early apoptosis was measured after indicated treatment using annexin V/PI-fluorescein isothiocyanate (AnnexinV/PI-FITC) (Vazyme, USA). Fluorescence signals were subsequently detected with excitation at 488 nm.

For staining of 5 mC, oocytes were fixed in 4% paraformaldehyde in PBS at room temperature for 30 min. They were then transferred to a membrane permeabilization solution (0.5% Triton X-100) for 30 min. After washing with PB1, oocytes were denatured with 4 N HCl for 10 min, neutralized with 100 mM Tris-HCl, pH 8.5, for 10 min and then incubated in PBS containing 0.05% Tween-20 at 4 °C overnight or at room temperature for 1 h. Subsequently, oocytes were incubated at room temperature for 1 h with a mouse anti-5 mC antibody (1:1000). After three washes in PB1, they were labelled with an Alexa Fluor 594 goat anti-mouse antibody (1:1000) at room temperature for 40 min. Then they were co-stained with Hoechst 33342 (10 μg/mL in PBS) for 10 min, then samples were analyzed by confocal microscopy.

For single staining of H3K27-me2 or H3K9-me2, oocytes were fixed in 4% paraformaldehyde in PBS at room temperature for 30 min. They were then transferred to membrane permeabilization solution (0.5% Triton X-100) for 20 min. After washing with PBS containing 1% BSA (PB1), oocytes were incubated with rabbit anti- H3K27me2 and H3K9me2 antibodies (1:200) overnight at 4 °C and then stained with Alexa Fluor 488 goat anti-rabbit antibody (1:500) at room temperature for 40 min. Then they were co-stained with Hoechst 33342 (10 μg/mL in PBS) for 10 min, then samples were analyzed by confocal microscopy.

Oocytes were then mounted on glass slides and examined with a Zeiss LSM 700 Meta scanning confocal microscope (Carl Zeiss, Inc.; Jena, Germany). At least 30 oocytes were examined for each experimental group. And the fluorescence intensity was analyzed by Image J.

### Transmission Electron Microscopy

Ovary tissue fragments were cut into 1 mm^3^ cubes, pre-fixed with 1% osmium tetroxide at RT for 1 h, fixed with 2.5% glutaraldehyde at 4 °C overnight, dehydrated using a graded series of ethanol and 100% acetone, and then embedded in epoxy resin. Ultrathin sections were cut, mounted on grids, and stained with uranyl acetate. These sections were observed with an H-7650 transmission electron microscope (Hitachi High-Technologies Corporation) and scanned with a Model S-3000N Scanning Electron Microscope. We get 10 sections per ovary, and analyzed the morphology of mitochondria in the sections.

### Statistical analysis

At least three replicates were used for each treatment with results are given as means ± SEM’s. Statistical comparisons were made using analysis of variance (ANOVA), followed by Duncan’s multiple comparisons test. A *p*-value of < 0.05 was considered significant.

For the analysis of fluorescence intensity, the samples of control oocytes and treated oocytes were mounted on the same glass slide. Image J software (NIH, Bethesda, MD, USA), was used to define a region of interest (ROI), and the average fluorescence intensity per unit area within the ROI was determined. And 30 oocytes were analyzed for each experiment. At least three replicates were used for each indicator.

## Additional Information

**How to cite this article**: Hou, Y.-J. *et al.* Both diet and gene mutation induced obesity affect oocyte quality in mice. *Sci. Rep.* **6**, 18858; doi: 10.1038/srep18858 (2016).

## Supplementary Material

Supplementary Information
