# Supplementary material for: Both diet and gene mutation induced obesity affect oocyte quality in mice
Source: Sci Rep. 2016 Jan 6;6:18858. doi: 10.1038/srep18858 (PMC4702149; doi:10.1038/srep18858)
Supplement: Supplementary Information [file srep18858-s1.doc]

**Both diet and gene mutation induced obesity affect oocyte quality in mice**

Yan-Jun Hou^1^, Cheng-Cheng Zhu^1^, Xing Duan^1^, Hong-Lin Liu^1^, Qiang Wang^2^, Shao-Chen Sun^1*^

Table S1:

Primer sequence

| Gene name | Primer sequence (5’-3’) | Size (bp) | AT1(°C) |
| --- | --- | --- | --- |
| Gapdh  (NM_008084.2) | F:ATGGTGAAGGTCGGTGTGAACG  R:CTCGCTCCTGGAAGATGGTGATG | 235 | 58 |
| Bcl-2  (NM_009741.4) | F:GTGGATGACTGAGTACCTGAACC  R:AGCCAGGAGAAATCAAACAGAG | 120 | 58 |
| Bak  (NM_007523.2) | F:TCGCCTCCAGCCTATTTAAG  R:TCCATCTGGCGATGTAATGA | 171 | 59 |
| SOD  (NM_013671.3) | F:ATGGTGGGGGACATATT  R:GAACCTTGGACTCCCACAGA | 166 | 57 |
| CAT  (NM_009804.2) | F:CCTCGTTCAGGATGTGGTTT  R:TCTGGTGATATCGTGGGTGA | 130 | 57-60 |
| GSH-Px  (NM_024198.3) | F:GTCCACCGTGTATGCCTTCT  R:TCTGCAGATCGTTCATCTCG | 152 | 57 |
